# Measurement of *S*-glutathionylated proteins by HPLC

**DOI:** 10.1007/s00726-021-03015-6

**Published:** 2021-06-15

**Authors:** Daniela Giustarini, Aldo Milzani, Isabella Dalle-Donne, Ranieri Rossi

**Affiliations:** 1 Department of Biotechnology, Chemistry and Pharmacy (Department of Excellence 2018-2022), Laboratory of Pharmacology and Toxicology, University of Siena, Via A. Moro 4, 53100 Siena, Italy; 2 Department of Biosciences (Department of Excellence 2018-2022), Università Degli Studi Di Milano, via Celoria 26, I-20133 Milan, Italy

**Keywords:** *S*-glutathionylation, HPLC, Whole blood, Cells, Protocols, Quantification

## Abstract

*S*-glutathionylated proteins (GSSP), i.e., protein-mixed disulfides with glutathione (GSH), are considered a suitable biomarker of oxidative stress. In fact, they occur within cells at low level and their concentration increases markedly under pro-oxidant conditions. Plasma is something different, since it is physiologically rich in *S*-thiolated proteins (RSSP), i.e., protein-mixed disulfides with various types of low molecular mass thiols (LMM-SH). However, albumin, which is largely the most abundant plasma protein, possesses a cysteine residue at position 34 that is mostly reduced (about 60%) under physiological conditions, but easily involved in the formation of additional RSSP in the presence of oxidants. The quantification of GSSP requires special attention to sample handling, since their level can be overestimated as a result of artefactual oxidation of GSH. We have developed the present protocol to avoid this methodological problem. Samples should be treated as soon as possible after their collection with the alkylating agent *N*-ethylmaleimide that masks –SH groups and prevents their oxidation. The GSH released from mixed disulfides by reduction with dithiothreitol is then labeled with the fluorescent probe monobromobimane and quantified by HPLC. The method can be applied to many different biological samples, comprising blood components, red blood cell plasma membrane, cultured cells, and solid organs from animal models.

## Introduction

Protein thiol groups (P-SH) can be present in biological systems in different redox forms, one of which is represented by mixed disulfides with low molecular mass thiols (LMM-SH), also defined as *S*-thiolated proteins (RSSP). More than 95% of RSSP within cells is represented by *S*-glutathionylated proteins (GSSP). The tripeptide glutathione (GSH) occurs at millimolar concentration within all mammalian cells (Hansen et al. 2009; Giustarini et al. 2017). The -SH group of the cysteine moiety confers to GSH a high reactivity towards a plethora of compounds. By reaction with oxidants, GSH can be converted to its disulfide forms, i.e., glutathione disulfide (GSSG) and GSSP. GSSP can form either by direct reaction with reactive oxygen species (ROS) or by reaction with secondary products of oxidative stress such as GSSG (Dalle-Donne et al. 2008). The susceptibility of P-SH to form mixed disulfides with LMM-SH depends on both the solvent accessibility of the thiol within the three-dimensional structure of the protein and the redox potential of the Cys residue.

GSSP have been investigated essentially for two main reasons. First of all, they are considered a good biomarker of oxidative stress in addition to GSSG. Inside mammalian cells, GSH exists almost totally in the reduced form, but both GSSG and GSSP can increase during oxidative perturbations (Schafer et al. 2001). Additionally, it is worth noting that, whereas GSSG is rapidly reduced by glutathione reductase, GSSP are less prone to enzymatic reduction (Giustarini et al. 2019). Second, *S*-glutathionylation is a post-translational modification of cysteine residues that can regulate protein functions by allosteric modification of their conformation. Therefore, *S*-glutathionylation of sensitive proteins can lead to a change in the activity or function of the oxidized protein, thus suggesting a role in physiological signaling (Grek et al. 2013). Moreover, since *S*-glutathionylation is a reversible process, it has been regarded as a protective mechanism against irreversible P-SH oxidation to sulfinic/sulfonic acids (Schafer et al. 2001**)**.

Thus, only accessible P-SH with high thiol-disulfide oxidation potential are likely to undergo *S*-glutathionylation under physio-pathological conditions. For example, *S*-glutathionylated hemoglobin has attracted interest as a clinical biomarker of oxidative stress. Human hemoglobin has an accessible Cys residue in position β93 (Garel et al. 1982) that occurs almost totally in the reduced form in healthy people but that it is supposed to be *S*-glutathionylated under specific pathological conditions, such as diabetes mellitus and Friederich ataxia (Niwa 2007; Piemonte et al. 2001). Albumin too possesses a free Cys in position 34 that can form mixed disulfides with LMM-SH (Sengupta et al. 2001). In contrast to the intracellular environment, the concentration of GSH in plasma is low (~ 2 µM) and other LMM-SH are present, namely cysteine, cysteinyl glycine, homocysteine, and γ-glutamylcysteine. Under physiological conditions, about 60%–70% of Cys34 in plasma albumin occurs as a free thiol, whereas the remaining Cys34 residues are involved in the formation of mixed disulfides with the physiological plasma LMM-SH (Sengupta et al. 2001; Turell et al. 2013). It is hypothesized that the percentage of *S*-thiolated albumin can increase in some diseases where oxidative stress has a role in the onset and/or progression of the disease (Candiano et al. 2009).

In vitro experiments with whole blood, red blood cells (RBCs), platelets, or cultured cells showed that GSSP increase rapidly under pro-oxidant conditions, i.e., after treatment with peroxides, diamide, disulfiram, or menadione (Rossi et al. 2001, 2006b; Giustarini et al. 2015). Moreover, GSSP concentration is found to be age-dependent in several rat tissues (Giustarini et al. 2011).

We must point out that this field of research is plagued by several pre-analytical artifacts, which usually lead to a large overestimation of GSSP. It is evident that, to better understand and define the physio-pathological role of GSSP, you need to pay attention to the methodological procedure used to detect them. We have demonstrated that -SH groups can be artefactually oxidized during sample handling in the pre-analytical step, thus raising the levels of both GSSG and GSSP (Rossi et al. 2002). This methodological problem should be particularly taken into consideration when the analysis of intracellular GSSP is carried out. In fact, the higher the real ratio GSH/GSSP, the higher the artificial increase in measured GSSP.

The protocol we describe and discuss here is commonly applied in our laboratory for GSSP quantification in cells and tissues. It has been developed to minimize all possible pre-analytical problems related to artificial oxidation of the -SH group with consequent overestimation of GSSP.

## Materials

### Chemicals and reagents

Acetonitrile (HPLC-grade): Sigma-Aldrich # 34851
Acivicin: Sigma-Aldrich # A2295
Boric acid : Sigma-Aldrich # B6768
Bovine serum albumin: Sigma-Aldrich #A3294
Bradford reagent: Supelco #B6916
Brij® L23 solution: Sigma-Aldrich # B4184
Disodium hydrogen phosphate: Sigma-Aldrich # 30412
Dithiothreitol: Millipore # 111474
Drabkin’s reagent : Sigma-Aldrich, # D 5941
Glacial acetic acid (HPLC-grade): EMD Millipore Chemicals # AX0074
*l*-Glutathione, reduced: Sigma-Aldrich # G4251
HPLC Zorbax Eclipse XDB-C18 column 4.6 × 150 mm, 5 μm: Agilent Technologies
Human hemoglobin : Sigma-Aldrich # H7379
Hydrochloric acid 37%: Sigma-Aldrich # 320331
Methanol (HPLC-grade): Sigma-Aldrich # 34860
Monobromobimane : Millipore # 596105
*N*-ethylmaleimide : Sigma-Aldrich # E1271
Phosphate buffer solution 1.0 M pH 7.4: Sigma-Aldrich # P3619
Potassium dihydrogen phosphate: Sigma-Aldrich, # 229806
l-Serine: Sigma-Aldrich # S4500
Sodium chloride: Sigma-Aldrich # S7653
Sodium hydroxide solution, 2 M: Fluka # 35254
Trichloroacetic acid: Sigma-Aldrich # T6399
Tripotassium EDTA: Fluka # 03664
Tris base: Sigma-Aldrich # T1503
Water (HPLC-grade): Sigma-Aldrich # 34877

### Equipment

The chromatographic separations reported in this article were performed using an HPLC Agilent 1100 series with fluorometric detector (Agilent Technologies). For each set of analyses, the column was first conditioned with 100% mobile phase B (HPLC-grade acetonitrile), 1.2 ml/min for at least 5 min, and the temperature equilibrated at 25 °C. Then, the mobile phase composition was changed to 94% phase A (sodium acetate 0.25% (v/v) pH 3.10) and 6% phase B. Run conditions: 0–5′ 6% phase B, 5’-10’ gradient until 10% phase B, 10’-10′50’’ 10% phase B. The injection volume was 10 µl. After each injection, at the end of the run, the column was flushed with 100% phase B for 2 min, and then, the system was re-equilibrated to the initial isocratic conditions before the subsequent injection. The signals were recorded setting excitation at 390 nm and emission at 480 nm. Retention times: Cys = 4.18 min, CysGly = 5.27 min, Hcys = 8.31 min, and GSH = 10.1 min. The retention factor for GSH is 4.

The determination of the protein content was performed by a UV–Vis spectrophotometer (Jasco, V-550).

### Small laboratory equipment

1. PD-10 desalting columns, bed volume 3.5 ml.

2. Semi-micro disposable cuvettes (Kartell, code 1938).

3. Microcentrifuge tubes, 1.5 ml (Eppendorf or equivalent).

4. Microcentrifuge (Mini Spin, Eppendorf).

5. Vortex 3 (IKA) with test tube inset.

6. Homogenizers (Potter Elvehjem P7859 or IKA Ultraturrax with S10 N-8G dispersing element).

7. HPLC vials (Agilent Technologies).

8. pH meter.

### Stock and working solutions

A. 310 mM NEM: dissolve 388 mg *N*-ethylmaleimide in 10 ml water.

B. 100 mM NaCl/20 mM phosphate buffer pH 7.4: dissolve 584 mg NaCl in 2 ml 1.0 M phosphate buffer solution and 98 ml water.

C. 20 mM Tris base: prepare 2 M Tris base by dissolving 24.2 g Tris in 100 ml water and then dilute it 1:100 in water.

D. 100 mM NaCl/20 mM Tris base: dissolve 584 mg NaCl in 1 ml 2 M Tris base and make the volume up to 100 ml with water.

E. 0.1 M phosphate buffer pH 6.5: dissolve 0.95 g KH_2_PO and 0.427 g Na_2_HPO_4_ in 80 ml water. Adjust the pH to 6.5 and make the volume up to 100 ml.

F. 5 mM phosphate buffer pH 6.5/NEM: prepare 10 ml buffer by mixing 0.5 ml 0.1 M phosphate buffer pH 6.5, 0.065 ml 310 mM NEM and 9.43 ml water.

G. PBS/NEM: mix 9.73 ml of normal saline solution (normal saline solution; 9 g of NaCl per liter), 0.065 ml 310 mM NEM and 0.2 ml of 1 M phosphate buffer pH 7.4.

H. TCA solutions: all the TCA solutions are prepared by diluting 60% w/v trichloroacetic acid (60 g TCA brought to a final volume of 100 ml with water).

I. Tris-BSAN: prepare 50 mM Tris buffer in water with serine/boric acid/acivicin/NEM (pH 8.0). Dissolve 3.03 g of Tris in 430 ml of water and add 0.62 g of boric acid, 105 mg of serine, 2 mg of acivicin and 50 ml of 310 mM NEM. Adjust the pH to 8.0 with 1 M HCl; adjust the volume to 500 ml with water.

L. 0.5 M Tris pH 7.8 containing 1 mM K_3_EDTA: dissolve 3 g Tris in 30 ml water. Add 22 mg K_3_EDTA, adjust the pH at 7.8, and make the volume up to 50 ml.

M. 10 mM DTT: dissolve 15.4 mg dithiothreitol in 10 ml water.

N. 40 mM mBrB: dissolve 10.8 mg monobromobimane in 1 ml methanol.

O. Mobile phase A (solution for HPLC): prepare 1 L of 0.25% (vol/vol) acetic acid by diluting 2.5 ml of glacial acetic acid with HPLC-grade water. Add a few drops of 2 M NaOH to bring the pH to 3.1.

## Protocol

### General aspects

**Fig. 1:**
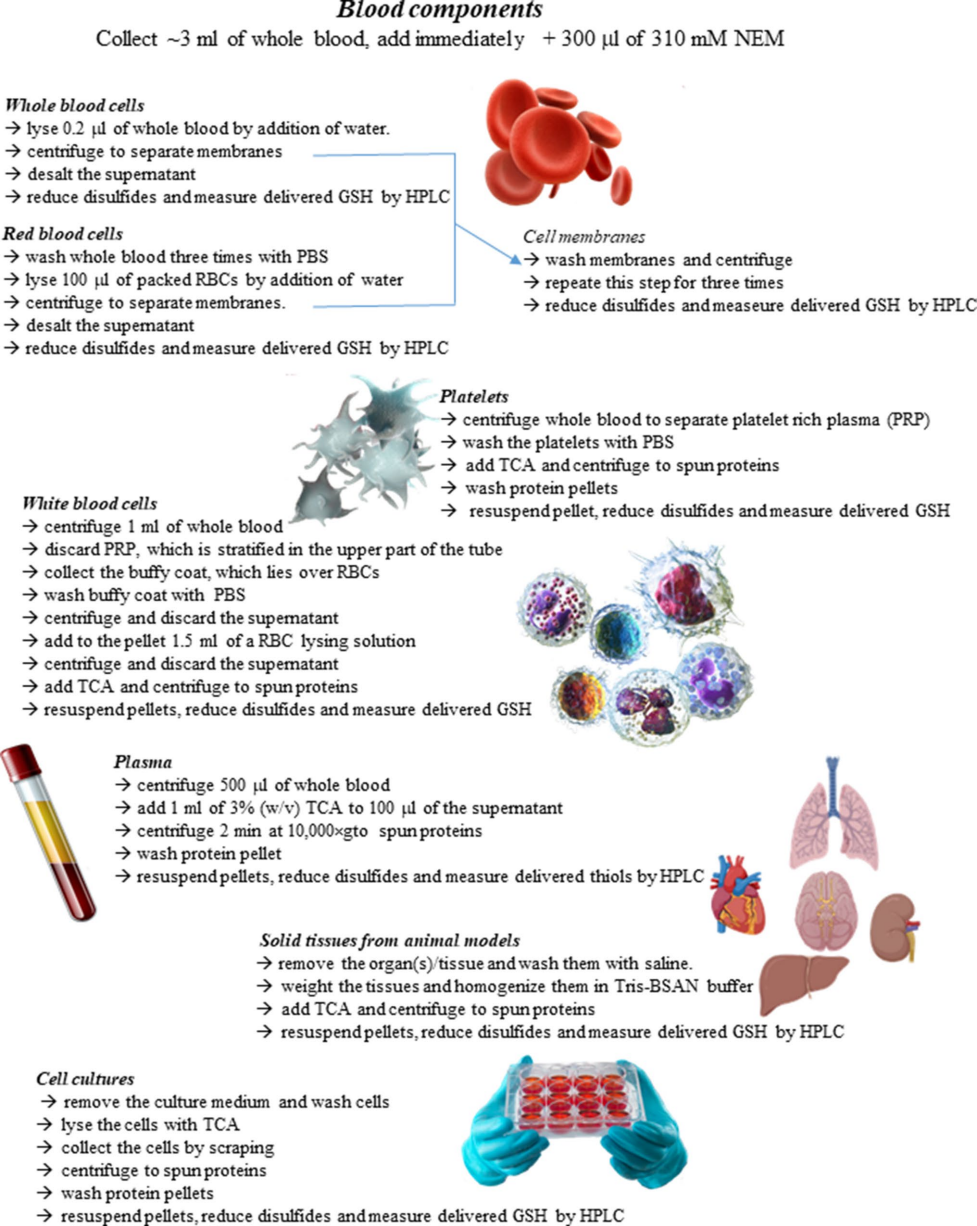
Schematic diagram of the main steps for RSSP detection in biological samples. The scheme describes the main pre-analytical steps required for RSSP detection in RBC plasma membrane, in the cytoplasm of blood components, cell cultures, and solid tissues, and in plasma. The delivered GSH (or LMM-SH for plasma samples) is then quantified by fluorometric HPLC

The protocol presented here has been developed to measure the total GSSP content in cells and tissues. It is based on the quantification of GSH released by reduction of GSSP. The main steps required for the application of the methods are shown in Fig. 1. A key step in GSSP analysis is the separation of proteins by the application of easy and time-saving methods. This step is usually carried out by acidification, followed by protein separation by centrifugation. However, we thoroughly demonstrated that the use of acids induces artificial oxidation of thiols to disulfides (Rossi et al. 2002). As a consequence, a fair amount of artificial GSSP can be formed during this phase. Even if this artifact can be limited by addition of chelating agents (e.g., EDTA) or the use of TCA instead of perchloric acid or metaphosphoric acid, we reported that also under these conditions, it cannot be avoided (Rossi et al. 2002). Since the cytoplasmic concentration of GSH is usually two or three orders of magnitude greater than that of GSSP, a minimal percentage of oxidation leading to the production of GSSP causes a massive artificial alteration of their levels. In a few words, the measured concentrations of intracellular GSSP are not physiological, but are mostly due to an artifact. We experienced this issue, in particular, when working with RBCs, probably due to the presence of iron and oxygen bound to hemoglobin, which during acidification formed reactive oxygen species (ROS) (Rossi et al. 2002). However, a treatment with an agent that can quickly block all free thiols before any further processing can solve the problem. We proved that the alkylating agent *N*-ethylmaleimide (NEM) is perfect for this purpose. This molecule was shown to easily cross membranes and to rapidly bind all present -SH groups, thus preventing their oxidation during sample handling (Rossi et al. 2002). Therefore, a common aspect of this protocol is the sample pre-treatment with NEM, which should be done as soon as possible after its collection. The excess of NEM can be easily removed by performing several washings of protein pellet under slight acidic conditions. At the end of this procedure, after the elimination of any trace of soluble GSH, a purified protein pellet is obtained, ready to undergo the proper treatment for the release of protein-bound GSH. An exception is the detection of *S*-glutathionylated hemoglobin (HbSSG) in RBCs. For detecting HbSSG, we separate the protein by gel-filtration to remove excess NEM, GSH, and GSSG at once (Giustarini et al. 2003). This procedure is preferred to the use of washed acid precipitated proteins as we noted that, after restoring neutral pH, the presence of a massive amount of denatured hemoglobin induces oxidation of the reducing agent used to detach GSH from GSSP, hampering a precise and accurate measurement of GSSP. Also, GSSP from RBC plasma membrane can be measured, because membrane separation is a preparative step performed before gel filtration. For this purpose, membranes are washed three times with PBS to eliminate GSH and GSSG before further processing.

The homogenization buffer for GSSP analyses in solid tissues must be slightly alkaline to speed up the alkylation of all -SH groups with NEM. However, the presence of γ-glutamyl transpeptidase (γGT) may present a challenge, because it can not only decompose soluble GSH but also GSSP. We obviate this potential drawback by including the γGT inhibitors borate, serine, and acivicin into the homogenization buffer.

For the cleavage of the S–S bridge, a solution of dithiothreitol (DTT) at slight alkaline pH is used. GSH released from GSSP is then labeled with monobromobimane (mBrB) and quantified by HPLC.

It is worthy of note that this procedure can detect not only GSSP but also protein-mixed disulfides with other LMM-SH. This is particularly useful when analyzing plasma samples, where several different LMM-SH are bound to albumin (Sengupta et al. 2001). Conversely, in the intracellular milieu, GSSP represent almost 100% of mixed disulfides present under both physiological conditions and oxidative stress conditions.

We report here, in detail, the procedure to measure GSSP in animal solid organs, but the same can be applied to human tissues. As for blood samples, the protocol for human samples is described, but it can be applied to blood samples from virtually all animal species too. Nevertheless, since rat hemoglobin can form precipitates when handled at low pH (Brunori et al. 1982), a specific procedure is described accordingly.

### Biological samples and preparative procedure

For the analyses in blood components collect ~ 3 ml of whole blood from the antecubital vein into K_3_EDTA vacutainers and add immediately 300 µl of 310 mM NEM. Invert gently the tubes for 10 times and then apply the procedure described below for the specific kind of blood component.

### Whole blood

Cytosolic whole blood GSSP: lyse 0.2 ml of whole blood by addition of three volumes of water.

Centrifuge at 15,000×*g* for 10 min to separate membranes. Desalt the supernatant with PD-10 columns equilibrated with 100 mM NaCl containing 20 mM Na^+^/K^+^ phosphate buffer pH 7.4. Eluted sample is ready for analysis or can be stored at − 80 °C for up to 6 months.

**!WARNING**: since rat hemoglobin crystallizes in hypoosmotic buffers, for cell lysis and desalting buffer a 20 mM Tris base solution and a 20 mM Tris base solution containing 100 mM NaCl are used, respectively.

Cell membrane GSSP from whole blood: centrifuge cell lysates as above described at 15,000×*g* for 10 min to separate membranes. Wash membranes with 5 mM Na^+^/K^+^ phosphate buffer pH 6.5 containing 2 mM NEM. Repeat this step three times suspending each time membrane pellet with a glass rod. Membrane samples are now ready for analysis or can be stored at − 80 °C for up to 6 months.

### Red blood cells

Cytosolic RBC GSSP: purify RBCs by washing three times 400 µl of whole blood with 1 ml of PBS containing 2 mM of NEM. Then lyse 100 µl of packed RBCs by addition of three volumes of water (or 20 mM Tris base for rat RBCs). Centrifuge at 15,000×*g* for 10 min to separate membranes. Desalt the supernatant with PD-10 columns equilibrated with 100 mM NaCl containing 20 mM Na^+^/K^+^ phosphate buffer pH 7.4 (or 20 mM Tris base for rat RBCs). Eluted sample is ready for analysis or can be stored at − 80 °C for up to 6 months.

Cell membrane GSSP: wash the membrane pellets obtained as above described with 5 mM Na^+^/K^+^ phosphate buffer pH 6.5 containing 2 mM NEM. Repeat this step three times suspending each time membrane pellet with a glass rod. Membrane samples are now ready for analysis or can be stored at − 80 °C for up to 6 months.

### Washed platelets

Centrifuge 1.5 ml of whole blood at 4200×*g* for 40 s to separate platelet-rich plasma (PRP), which is stratified in the upper part of the tube. Collect PRP carefully avoiding the buffy coat, which lies over RBCs. Wash three times the platelets with 1 ml PBS containing 2 mM NEM and, after the third washing, discard the supernatant and add 1 ml of 3% (w/v) TCA. Mix well and centrifuge for 2 min at 14,000×*g*. Repeat this step for three times resuspending with a glass rod. Finally, discard the supernatant. Samples are now ready for analysis or can be stored at − 80 °C for up 6 months.

### White blood cells

Centrifuge 1 ml of whole blood at 4200×*g* for 40 s to separate PRP, which is stratified in the upper part of the tube. Collect the buffy coat, which lies over RBCs. Add 1 ml of PBS containing 2 mM NEM and centrifuge 30 s at 10,000×*g*. Discard the supernatant and add to the pellet 1.5 ml of an RBC lysing solution (0.1 M sodium chloride in 0.05 M Na^+^/K^+^ phosphate buffer pH 7.4). Allow to settle for 30 min, then centrifuge for 5 min at 10,000×*g* and discard the supernatant. This step eliminates RBCs collected along with the buffy coat. Add 1 ml of 3% (w/v) TCA, mix well and centrifuge for 2 min at 14,000×*g*. Repeat this step for three times resuspending with a glass rod each time. Discard the supernatant. Samples are now ready for analysis or can be stored at − 80 °C for up 6 months.

### Plasma

Centrifuge 500 µl of whole blood at 10,000×*g* for 20 s. Add to 100 µl of the supernatant 1 ml of 3% (w/v) TCA. Centrifuge 2 min at 10,000×*g*. Discard the supernatant and wash three times with 1 ml of 3% (w/v) TCA resuspending each time the pellet with a glass rod. Finally, discard the supernatant. Samples are now ready for analysis or can be stored at − 80 °C for up 6 months.

### Cell cultures

Remove the culture medium and wash cells twice (1 min each) at room temperature with PBS/NEM. Lyse the cells by treatment with 1 ml of 4% (w/v) TCA. Collect the cells by scraping. Mix well and centrifuge for 2 min at 14,000×*g*. Discard the supernatant and repeat this step for three times resuspending with a glass rod each time. Finally, discard the supernatant. Samples are ready for analysis or can be stored at − 80 °C for up to 6 months.

### Solid tissues from animal models

Collect the blood from the anesthetized animal through the abdominal aorta, and during the withdrawal infuse 10 ml of saline containing 5 mM NEM through the inferior cava vein. After that collect and rapidly wash the organ(s)/tissue with ice-cold saline.

Weight the tissues and homogenize them 1: 10 (w/v) in Tris-BSAN buffer, wait for 1 min and then acidify by 1:1 addition of 10% (w/v) TCA. Mix well and centrifuge for 2 min at 14,000×*g*. Repeat this step for three times resuspending with a glass rod. Finally, discard the supernatant. Samples are ready for analysis or can be stored at − 80 °C for up to 6 months.

### Validation of the method

**Fig. 2:**
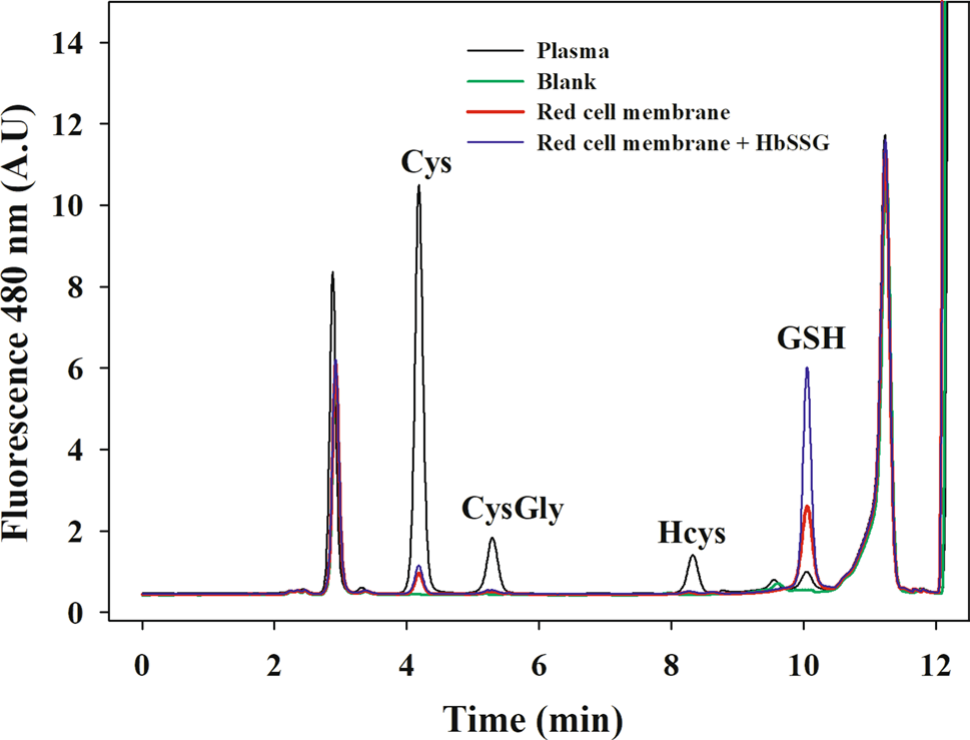
Chromatogram showing the RSSP content in some biological samples. LMM-SH released by dithiothreitol cleavage are labeled with monobromobimane and separated by HPLC. A typical chromatogram is shown for GSSP in red blood cell plasma membrane and for RSSP in plasma. An exemplificative chromatogram obtained for RBC membrane samples added with a standard solution of *S*-thiolated hemoglobin (HbSSP, 2 nmol/g Hb) is reported too. The blank chromatogram refers to the analysis of a sample prepared with all the reagents but without the RBC membranes

An exemplificative chromatogram obtained by the analysis of RSSP in RBCs’ membrane and plasma is reported in Fig. 2. The accuracy and the precision of the procedure were tested by addition of standard solutions of HbSSG prepared as previously described (Giustarini et al. 2015) and analyzed on three separate days. The precision of the method was expressed as the relative standard deviation (RSD) and was always well below 5%. The accuracy was expressed as relative error (RE) [(mean observed concentration − spiked concentration)/(spiked concentration)] × 100% and ranged from − 1.35 to 2.34% (intraday accuracy) and from − 2.84 to 1.63% (interday accuracy).

The quantification limit of the method is 5 pmol/mg protein.

### RSSP reduction and derivatization for HPLC analysis

A. Plasma, washed platelets, white blood cells, cultured cells, solid tissues, whole blood, and RBC membranes.

Timing: 30 min.

1. Resuspend pellet with 0.4 ml of 0.075 M TRIS base containing 1 mM EDTA and 1 mM DTT.

2. Put the samples in a gyratory shaker for 20 min.

3. Deproteinize 0.1 ml sample by acidification with 40 µl 60% (w/v) TCA.

4. Centrifuge 2 min at 10,000×*g*.

5. React 0.1 ml supernatant with 5 µl of 40 mM mBrB and 20 µl 2 M TRIS base for 10 min in the dark.

6. Add 5 µl 37% (v/v) HCl.

7. Centrifuge 2 min at 10,000×*g*.

Samples are ready for HPLC analysis.

B. Whole blood and RBC cytosolic fractions.

Timing: 30 min.

1. React 0.1 ml protein fraction separated by gel filtration or purified membranes with 5 µl of 10 mM DTT for 15 min.

2. Add 5 µl of 40 mM mBrB.

3. Place the sample in the dark for 15 min.

4. Deproteinize the sample by addition of 10 µl of 60% (w/v) TCA.

Samples are ready for HPLC analysis.

### Quantification by HPLC

Timing: about 15 min per sample.

Chromatographic separation is performed on an Agilent series 1100 HPLC device with a Zorbax Eclipse XDB-C18 column and fluorometric detector.

Determination of concentration is performed by running standard concentrations of GSH (or other LMM-SH for plasma analyses).

### Normalization for protein content

The normalization for protein content is carried out for whole blood, RBCs, membrane, white blood cells, and cultured cells analyses.

The concentration of hemoglobin in RBCs is obtained by colorimetric determination at 540 nm using the Drabkin’s reagent. The reagent is prepared according to the manufacturer’s instructions by dissolving it with Brij^®^ L23 solution. Human hemoglobin is used as a standard.

The protein concentration in RBC membranes, cultured cells, white blood cells, and whole blood is determined by colorimetric reaction with the Bradford reagent (Bradford 1979). Protein pellets are resuspended in 0.1 N NaOH and mixed in a rotatory shaker to facilitate protein dissolving. An aliquot of the sample (typically 10 µl) is reacted with the Bradford reagent and analyzed at 595 nm wavelength. Bovine serum albumin is used as a standard.

Platelets values are normalized for cell count and solid tissue values for tissue weight.

## Biomedical applications

### RSSP in RBCs

**Fig. 3:**
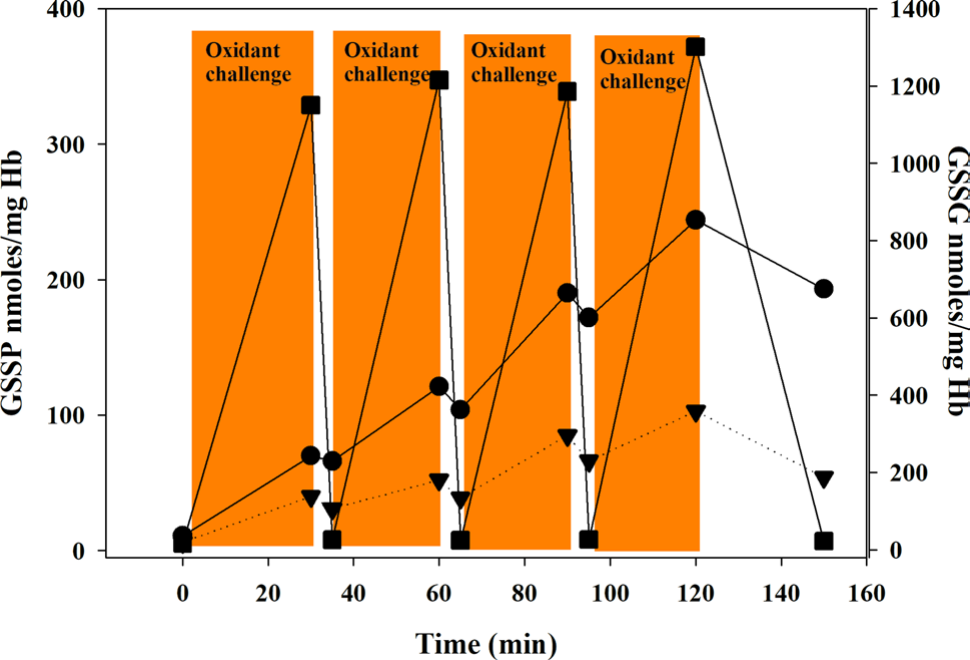
Cytoplasmic and membrane GSSP and GSSG in RBCs treated with *t*-BOOH. RBCs at a 10% hematocrit were treated by a slow flux of *t-*BOOH for 120 min (flux rate, 0.7 μmol/min) but with a cyclic 4-min time without exposure to the oxidant. At the indicated times, aliquots of sample were collected for HbSSG (triangle), membrane GSSP (circle), and GSSG (square) determination both from the treatment vessel and from a collection point not exposed to the oxidant (4 min after). Times of analysis from the treatment vessel: 0, 30, 60, 90, and 120 min. Times of analysis of samples not exposed to the oxidants: 34, 64, 94, 150 min. Data are the mean of four separate experiments (Giustarini et al. 2019)

The protocol described in this manuscript for the detection of GSSP in RBCs as biomarkers of oxidative stress was applied in our laboratory to human and rat samples. In Table 1, the reference values for both cytoplasmic and membrane GSSP are reported. It is evident that the basal levels of intracellular GSSP are usually very low, being about 2–3 orders of magnitude lower than cytoplasmic GSH concentration (Giustarini et al. 2003; Khazim et al. 2013). These values largely differ from those reported by other research groups (Table 2). We believe that this discrepancy is driven primarily by lack of standardization for properly addressing the technical challenge of GSH oxidation during sample collection. This makes it harder to compare results and slows down the advancement of knowledge in the field of research. Membrane GSSP occur at low levels in healthy people too (Giustarini et al. 2003). However, we have recently demonstrated that membrane GSSP concentration can increase more than that of cytoplasmic GSSP in RBCs under slight and intermittent oxidative conditions, which can mimic the physiological ones. In Fig. 3, the in vitro treatment of RBCs with a cyclic low concentration of *tert*-butyl hydroperoxide (*t*-BOOH) slowly delivered with a particular device is reported. *t*-BOOH induced a stepwise increase of both HbSSG and membrane GSSPs; nevertheless, membrane GSSP appear to be less influenced by the absence of the peroxide. As a matter of fact, membrane GSSP decreased more slowly than HbSSG when *t*-BOOH infusion was stopped, suggesting that membrane GSSP decreased slowly when pro-oxidant conditions are absent. It should also be noted that the oxidant-induced membrane GSSP were more abundant than HbSSG at all the analyzed times (Giustarini et al. 2019). Interestingly, as reported in Fig. 3, under slight intermittent oxidative stress GSSG appears to rise and decrease much faster than both membrane and cytosolic GSSP, thus suggesting that HbSSG and even more membrane GSSP are stable biomarkers of oxidative stress, which are likely less affected than GSSG by either temporary variation of oxidant stimulus or sample manipulation.

**Table 1:** Values for GSSP measured in different blood components

Sample	Red blood cells		References
	Cytoplasmic nmol/g Hb	Membrane nmol/g protein	
Healthy humans (*n* = 12)	9.90 ± 3.02	0.736 ± 0.159	Giustarini et al. (2003)
Healthy humans (*n* = 21)	13 ± 0.3		Khazim et al. (2013)
HD patients (*n* = 33)	20 ± 0.5		Khazim et al. (2013)
Rat (*n* = 10)	10.5 ± 2.57	0.682 ± 0.259	Giustarini et al. (2003)
Platelets			
Healthy humans (*n* = 5)	0.046 ± 007 nmol/10^9^ plt		Giustarini et al. (2016)
White blood cells (cytoplasmic + membrane)			
Healthy humans (*n* = 10)	37.7 ± 12.4 nmol/10^6^ cells		Unpublished results

Data are the mean ± SD

**Table 2:** Levels of HbSSP measured in human blood from healthy people

nmol/mg Hb	Age	Reference
1050 ± 660 (*n = *15)	49 ± 7.6	Mandal et al. (2007)
1230 ± 100 (*n = *20)	–	Naito et al. (1999)
1000 ± 530 (*n = *20)	47 ± 10	Takayama et al. (2001)
1287 (*n = *41)	55.7 ± 12.8	Schepens et al. (2006)
880 ± 300	43	Muscat et al. (2004)
200 *(n = *9)	–	Al-Abed et al. (2001)
166 ± 450 (*n = *30)	11.4 ± 3.73	Pastore et al. (2012)
4380 ± 2010	23.4 ± 6.5	Chen et al. (2014)

Data are expressed as *nmoles*/*g* *Hb* normalized for Hb content for comparison by considering a mean concentration of 150 mg/ml whole blood

The results of a clinical study, where the levels of HbSSG were measured in erythrocytes of healthy people (*n* = 21) and in maintenance hemodialysis (HD) patients (*n* = 33), are also reported in Table 1. We found that GSSP were 46% higher in HD patients than in age-matched healthy controls. It is worthy of note that a significant increase in cysteinylated hemoglobin (CySSHb) in HD patients was also observed (38.3 vs 11.5 pmol/mg Hb; *p* < 0.001) and that RBCs from HD patients contained twice as much CySSHb than HbSSG (Khazim et al. 2013).

**Fig. 4:**
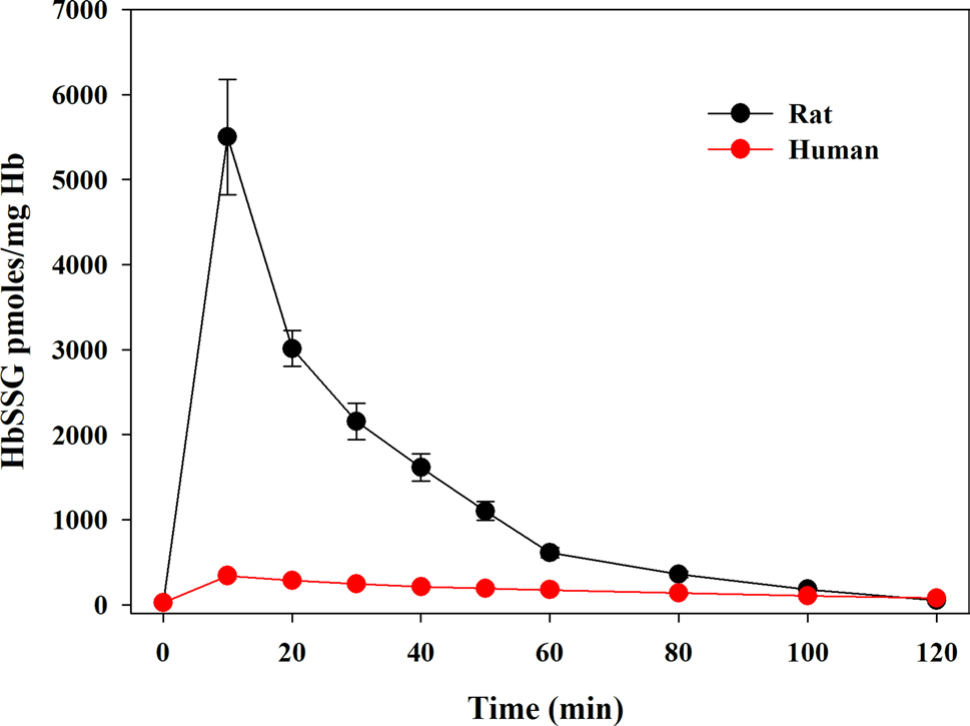
Cytoplasmic HbSSG in human and rat RBCs treated with *t*-BOOH. RBCs were exposed to 1.5 mM *t-*BOOH (final concentration). At the indicated times, *S*-glutathionylated hemoglobin was measured in whole RBC lysates. Times of analysis: 0, 10, 20, 30, 40, 50, 60, 80, 100, and 120 min. Data represent the means ± SD of three separate experiments (Colombo et al. 2010)

Rat hemoglobin possesses some extra-reactive –SH groups that make this protein exceptionally susceptible to *S*-glutathionylation under pro-oxidative conditions (Rossi et al. 2001). As shown in Fig. 4, an in vitro treatment of RBCs with *t*-BOOH induced a dramatic increase in HbSSG in rat samples but not in human ones. This particular susceptibility was observed also in some mice strains (Giustarini et al. 2006). *S*-glutathionylation of hemoglobin does not interfere with the allosteric capacity of hemoglobin to bind and transport oxygen. It can be thus interpreted as an extra antioxidant defense that RBCs of these animal species can exploit.

### GSSP in platelets and white blood cells

**Fig. 5:**
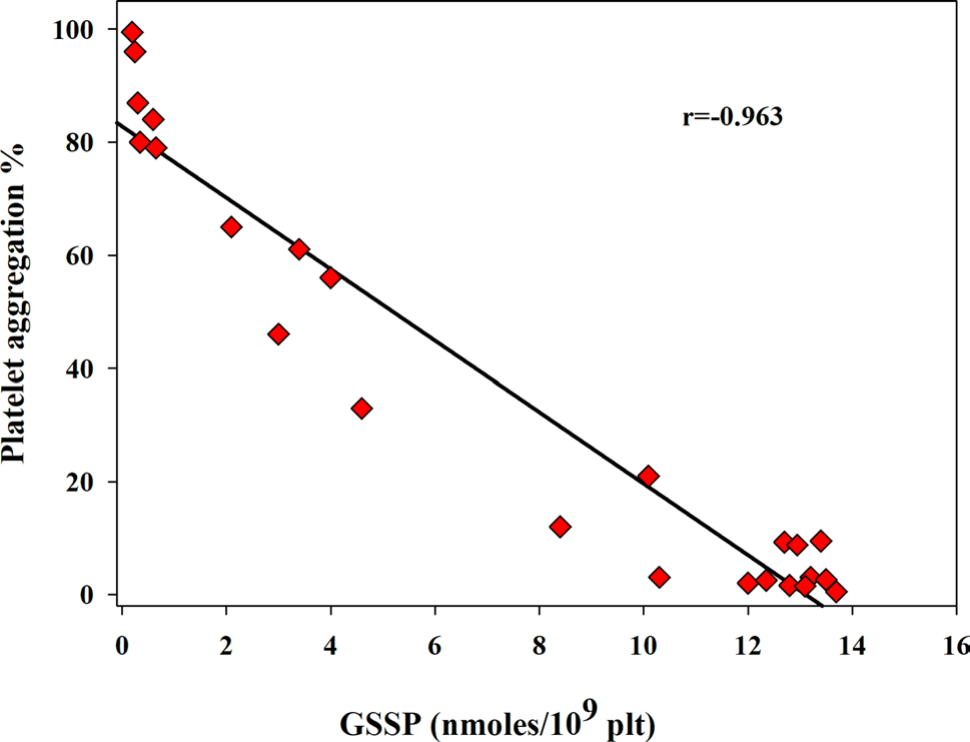
Correlation between GSSP concentration and the percentage of platelet aggregation. Human platelets were exposed to 0.1, 0.3, and 1 mM disulfiram. Platelet aggregation was initiated by the addition of ADP (10 µM, final concentration) and was monitored for 10 min by continuous recording of light transmission in a platelet aggregometer (Rossi et al. 2006a)

Basal levels of GSSP in platelets from healthy humans measured with this protocol were 0.046 ± 007 nmol/10^9^ plt (Table 1). Treatment of platelets with disulfiram (a drug used to prevent alcoholism that has a reactive disulfide bridge in his structure) induced a dose-dependent increase in GSSP. When platelets were exposed to 1 mM disulfiram, irreversible formation of GSSP occurred, with all cytosolic GSH bound to proteins to form GSSP. Instead, at lower concentrations of the drug, the recovery of both GSH and GSSP within 1 h was observed. Interestingly, the concentration of GSSP was shown to greatly affect platelet aggregation, indicating that P-SH have a key role in platelet activity. In fact, ADP-induced platelet aggregation was found to be inversely correlated with GSSP Fig. 5 (Rossi et al. 2006b). Actin can play a role in this process. Indeed, we found that, after treatment with disulfiram, actin is largely *S*-glutathionylated, probably because it has a solvent-exposed and extremely reactive Cys residue at position 374 that is susceptible to *S*-glutathionylation (Dalle-Donne et al. 2003).

Basal levels of GSSP in white blood cells were rather difficult to measure because, after treatment with NEM (which is necessary to avoid artifacts), it is not easy to separate them from other blood components. As a matter of fact, we have not been able so far to separate lymphocytes and polymorphonuclear cells using common techniques (separation based on distinct density differences) after treatment with NEM. Therefore, we can report only levels of GSSP on whole blood leukocytes.

### RSSP in cultured cells and solid tissues

We recently applied the present protocol to measure GSSP levels in several cell lines (Giustarini et al. 2015). Table 3 shows that GSSP levels in different cell lines fall in the range 14.0 ± 3.3 pmol/mg of protein for human prostate cancer-derived bicalutamide-resistant cells (LNCap-Rbic) to 140 ± 26 pmol/mg of protein in human glioblastoma-derived cells (U87). In all the analyzed samples, the treatment with the oxidant drug disulfiram induced a remarkable dose-dependent increase in GSSP even at very low concentrations (20 µM or even less). The levels we measured are in agreement with those measured by Hansen et al. (Hansen et al. 2009), who found very low levels of GSSP also in HEK and HeLa cells, with values of a factor of 1000 below the value of total protein disulfides (i.e., PSSP + GSSP). Analogously, very low levels of GSSP (i.e., 57 ± 42 nmol/mg protein) were measured in rat liver by means of LC–MS/MS (Bukowski et al. 2015). Levels of GSSP in solid tissues from rats at different ages are reported in Table 4. These values well agree with those reported by other research groups (Bukowski et al. 2015; Arambasic et al. 2013) that found 57 ± 42 nmol/mg protein GSSP in rat liver and 1.75 µM GSSP in rat kidneys, respectively. GSSP were found to be significantly increased in some organs (lung, heart, spleen, brain) with aging (Giustarini et al. 2011), thus supporting a role of free radicals in this physio-pathological process (Viña et al. 2007).

**Table 3:** Levels of GSSP in several cell lines under basal conditions and after a 15-min treatment with the pro-oxidant drug disulfiram

Cell line	Control	20 µM Disulfiram
BAEC^a^	110 ± 17	2378 ± 67
HUVEC	24.3 ± 1.2	476 ± 54
Panc-1	87.1 ± 10	1423 ± 96
NT2-D1	27.8 ± 3.0	86.3 ± 11.2
A549	50.1 ± 7.5	96.9 ± 5.5
RD	107 ± 3	238 ± 31
HEK	47.3 ± 3.6	284 ± 19
T98	330 ± 41	379 ± 22
HaCaT	20.6 ± 1.4	85.1 ± 6.3
U87	140 ± 26	427 ± 16
IMR-90	28.3 ± 3.7	493 ± 75
BRC-230	26.4 ± 2.4	269 ± 32
MCF-7	33.9 ± 3.0	186 ± 11
A 375	233 ± 13	11,050 ± 715
LNCaP	20.7 ± 7.1	45.9 ± 1.1
LNCaP-Rbic	14.0 ± 3.3	32.2 ± 2.6

Data (expressed as pmoles/mg protein) are the mean ± SD, *n = *3

^a^*BAEC* bovine aortic endothelial cells, *HUVEC* human umbilical vein endothelial cells, *Panc*-*1* human pancreatic carcinoma-derived cells, *NT2*-*D1* human pluripotent embryonal carcinoma-derived cells, *A549* human lung carcinoma-derived cells, *RD* human rhabdomyosarcoma-derived cells, *HEK* *293* human embryonic kidney-derived cells, *T98G* human brain glioblastoma-derived cells, *HaCaT* human spontaneously immortalized keratinocyte cells, *U87* human glioblastoma-derived cells, *IMR-90* human embryonic lung-derived fibroblasts, *BRC*-*230* human breast cancer-derived cells, *MCF*-*7* human breast cancer-derived cells, *A375* human melanoma-derived cells, *LNCaP* human prostate cancer-derived cells, *LNCaP*-*Rbic* bicalutamide-resistant cells derived from LNCaP

**Table 4:** Values for GSSP in solid rat tissues at different ages

Tissue	3 months	9 months	20 months
Liver	4.44 ± 0.80	4.54 ± 0.60	5.65 ± 0.60
Kidney	2.14 ± 0.30	1.76 ± 0.11	1.76 ± 0.61
Lung	2.12 ± 0.32	1.89 ± 0.24	2.81 ± 0.62*^,#^
Heart	1.06 ± 0.07	1.13 ± 0.11	1.72 ± 0.60**^, #^
Spleen	6.45 ± 0.53	5.09 ± 0.88	8.68 ± 0.60**^, ##^
Testis	1.48 ± 0.20	1.51 ± 0.05	0.90 ± 0.61
Brain	5.81 ± 0.42	5.96 ± 0.77	8.08 ± 0.61*^,#^

Data are expressed as nmol/g wet tissue and are the mean ± SD, *n = *4 for each study group. *p < 0.05 vs 3-month-old rats; **p < 0.01 vs 3-month-old rats; #*p* < 0.05 vs 9-month-old rats; ##*p* < 0.01 vs 9-month-old rats

### RSSP in plasma

Plasma content of RSSP is very different from the intracellular one both in terms of concentration and species of LMM-SH bound to proteins. In fact, plasma is quite poor in antioxidants and glutathione reductase is present only in traces, which probably derive from cellular disruption. As for thiols, plasma is characteristically rich in their disulfide forms and contains not only GSSP but also *S*-cysteinylated (CySSP) and *S*-homocysteinylated (HcySSP) proteins. In addition, also protein-mixed disulfides with cysteinyl glycine (CyGlySSP) and γ-glutamylcysteine (γ-GluCySSP) are generally found (Turell et al. 2013).

The values for different RSSP measured in 22 healthy controls with the present protocol are reported in Table 5 (Fanti et al. 2015). These values are in agreement with those reported by others (Grintzalis et al. 2014). For some of these (CySSP, CyGlySSP, HcySSP), we observed a significant increase in maintenance HD patients. CySSP concentration resulted to be directly related to plasma neutrophil gelatinase-associated lipocalin (NGAL) in maintenance HD patients, suggesting functional coupling of thiol stress and acute-phase response in uremia. High circulating levels of NGAL are only in part the consequence of impaired renal elimination of the protein, as they also result from increased systemic production in response to chronic kidney disease-related inflammation and possibly to iron supplementation (Bolignano et al. 2009; Chakraborty et al. 2012).

**Table 5:** Values for RSSP in plasma of healthy people and in maintenance hemodialysis (MHD) patients

Sample	CySSP^a^	CyGlySSP	HcySSP	γ-GluCySSP	GSSP
Healthy humans (*n = *24)	163 (150–195)	15.0 (13.3–18.1)	8.23 (6.45–9.96)	1.55 (1.31–1.71)	3.11 (2.66–3.38)
MHD (*n = *71)	216** (182–254)	21.0** (16.5–25.5)	18.5** (14.9–23.4)	1.51 (1.22–1.78)	3.01 (2.07–3.91)

Data are the median and are expressed as μM. **p < 0.001 vs healthy humans

^a^*CySSP* protein-mixed disulfides with cysteine, *CyGlySSP* protein-mixed disulfides with cysteinyl glycine, *HcySSP* protein-mixed disulfide with homocysteine, γ-*GluCySSP* protein-mixed disulfides with γ-glutamylcysteine, *GSSP* protein-mixed disulfides with GSH

## Conclusion

Post-translational modification of thiol groups is considered to be one of the main processes involved in redox signaling. Given its reversibility, *S*-glutathionylation is supposed to have an important role for the redox switching of proteins, thus altering their function (Mieyal and Chock 2012). Additionally, *S*-glutathionylated proteins are currently investigated as powerful biomarkers of oxidative stress. Nevertheless, their analysis is not easy, since their concentration can be overestimated as a result of the oxidation of thiols during the pre-analytical step. Here, we describe a protocol that avoids virtually all methodological problems by protecting the -SH group from its artificial oxidation. By applying this procedure, we have been able to demonstrate that the basal levels of GSSP are very low in most biological samples, with the exception of the extracellular compartment. Our values are similar to those reported by other research groups for some tissues (e.g., plasma, rat liver, and kidney) but are significantly lower for HbSSG. It is possible that blood is particularly prone to GSH auto-oxidation by virtue of the presence of oxygen and iron in the heme group. Therefore, the need to spike the blood samples as soon as possible with the suitable GSH blocking agent is particularly evident. We demonstrated that GSSP represent a sensitive biomarker of oxidative stress, since their concentration increases significantly also at low concentrations of oxidants (Rossi et al. 2006a; Giustarini et al. 2019). However, this sensitivity dramatically decreases when GSSP are overestimated. The protocol can be applied to all biological samples and the quantification by HPLC with fluorometric detector allows the discrimination among the different LMM-SH involved in the RSSP formation. Obviously, this method is characterized by pros and cons. The main pro is that it is quantitative and can estimate with good precision and accuracy the total amount of GSSP in a biological sample. The main con is that it cannot discriminate among the various GSSP, and thus, it is impossible to identify every single protein undergoing *S*-glutathionylation.

## Abbreviations

CyGlySSP: Protein mixed disulfides with cysteinyl glycine
CySSHb: *S*-Cysteinylated hemoglobin
CySSP: *S*-Cysteinylated proteins
DTT: Dithiothreitol
γ-GluCySSP: Protein mixed disulfides with γ-glutamylcysteine
γ GT: γ-Glutamyl transpeptidase
GSH: Glutathione
GSSG: Glutathione disulfide
GSSP: *S*-Glutathionylated proteins
HbSSG: *S*-Glutathionylated hemoglobin
HcySSP: *S*-Homocysteinylated proteins
HD: Hemodialysis
LMM-SH: Low molecular mass thiols
mBrB: Monobromobimane
NEM: *N*-Ethylmaleimide
NGAL: Neutrophil gelatinase-associated lipocalin
PBS: Phosphate-buffered solution
PRP: Platelet-rich plasma
P-SH: Protein thiol groups
RBCs: Red blood cells
ROS: Reactive oxygen species
RSSP: *S*-Thiolated proteins
*t-*BOOH: *tert*-Butyl hydroperoxide
TCA: Trichloroacetic acid
